# A Mathematical Model for the Variation of Cerebral Electrical Conductivity and the Amount of β-Amyloid Protein Values Due to Alzheimer’s Disease

**DOI:** 10.3390/brainsci15121313

**Published:** 2025-12-09

**Authors:** Emmanouil Perakis, Panagiotis Vlamos

**Affiliations:** Bioinformatics and Human Electrophysiology Laboratory, Department of Informatics, Ionian University, 49100 Corfu, Greece; c19pera@ionio.gr

**Keywords:** Alzheimer’s disease, β-amyloid, brain conductivity, variation, mathematical model

## Abstract

**Background/Objectives:** This study presents a time-dependent mathematical model that describes how progressive amyloid-β (Aβ) accumulation drives the gradual decline of cerebral electrical conductivity during Alzheimer’s disease (AD). **Methods:** The formulation captures the coupled evolution of molecular burden and electrophysiological function through a pair of interconnected dynamical processes, enabling a mechanistic link between early biochemical alterations and large-scale neural degradation. **Results:** Simulations reveal a characteristic pattern in which Aβ levels rise steadily toward a pathological plateau, while conductivity follows a delayed but persistent downward trajectory that stabilizes at an impaired state consistent with advanced neurodegeneration. The model reproduces key phenomena reported in experimental and clinical studies, including the slow, irreversible reduction in cortical conductivity and the strong inverse relationship between amyloid burden and electrophysiological integrity. **Conclusions:** Although intentionally minimal, the framework offers a tractable basis for interpreting disease progression and can be extended to incorporate additional pathological pathways such as tau aggregation, inflammatory responses, or spatial heterogeneity. By providing a compact yet biologically meaningful representation of the interplay between molecular pathology and electrical dysfunction, the model supports the development of computational biomarkers and contributes to a more integrated understanding of AD progression.

## 1. Introduction

AD is a chronic, progressive neurodegenerative disorder marked by neuronal loss, synaptic dysfunction, and the gradual breakdown of both cortical and subcortical networks. Its defining pathological features include the extracellular accumulation of Aβ plaques, the intracellular aggregation of hyperphosphorylated tau, and a measurable decline in brain electrical conductivity—processes that collectively disrupt neural communication and impair cognitive performance [1]. Among these interacting mechanisms, Aβ plays a central initiating role. Imbalances between its production and clearance lead to extracellular buildup, the formation of insoluble aggregates, and subsequent induction of oxidative stress and inflammatory responses [2,3,4,5,6,7]. These molecular events progressively alter the dielectric properties of neural tissue, ultimately producing the reductions in conductivity documented across electrophysiological and imaging studies [8,9].

Mathematical modelling provides a structured way to quantify these interdependent processes and explore how molecular disturbances propagate to macroscopic functional decline [10,11,12,13,14,15,16,17]. By expressing biological mechanisms in the form of dynamical systems, such models enable the identification of factors that govern disease evolution and make it possible to investigate scenarios that would be difficult—or ethically constrained—to test experimentally [11,18]. In this study, a streamlined yet physiologically meaningful formulation is introduced to capture the interplay between two core components of AD pathology: the temporal accumulation of Aβ and the associated deterioration of cerebral electrical conductivity. While previous work has explored more extensive multivariable models incorporating tau propagation or inflammatory pathways [9], the present framework concentrates on isolating the direct and measurable relationship between amyloid burden and electrical decline.

The model employs two coupled dynamical expressions: one describing the gradual buildup of Aβ driven by production and clearance processes, and another linking amyloid burden to conductivity loss. Despite its intentional simplicity, the formulation reproduces key features of AD progression, including the long-term rise in Aβ concentration, the characteristic transition from initial stability to conductivity decline, and the eventual convergence toward a chronically impaired state. These behaviours reflect patterns consistently observed in experimental and clinical data, underscoring both the biological relevance and interpretative value of the proposed approach.

## 2. Variation in the Amount of Aβ in Brain Tissue

The accumulation of Aβ in neural tissue is one of the earliest and most defining biochemical events in AD. Overproduction or reduced clearance of Aβ peptides leads to extracellular aggregation, plaque formation, and progressive disruption of neural communication [3,4,18]. The slow but persistent imbalance between production and degradation initiates a self-sustaining cascade of oxidative stress and synaptic toxicity that underlies the clinical progression of the disease [2,19].

To describe the temporal evolution of Aβ, the model considers a first-order linear o.d.es that expresses the balance between a constant production term and an effective degradation process. Let A(t) denote the total Aβ concentration at time t. Then,

(1) $$\frac{dA}{dt}=A_{c}-aA(t)$$

where A_c_ represents the constant rate of Aβ production and a is the effective degradation coefficient. The analytical solution of this equation is

(2) $$A\left(t\right)=\frac{A_{c}}{a}\left(1-e^{-at}\right)+A_{0}e^{-at}$$

where $A_{0}\equiv A(0)$ is the initial concentration of Aβ at t=0. This expression shows that Aβ concentration increases exponentially and asymptotically approaches a steady-state level $A_{s}=\frac{A_{c}}{a}$. The equilibrium represents a dynamic balance between production and clearance. When the degradation coefficient a is low, the convergence toward $A_{s}$ is slower and the steady-state value is higher—conditions that mirror the pathological accumulation observed in AD brains. Conversely, efficient enzymatic degradation or enhanced microglial activity (large a) prevents Aβ from reaching toxic levels.

**Figure 1:**
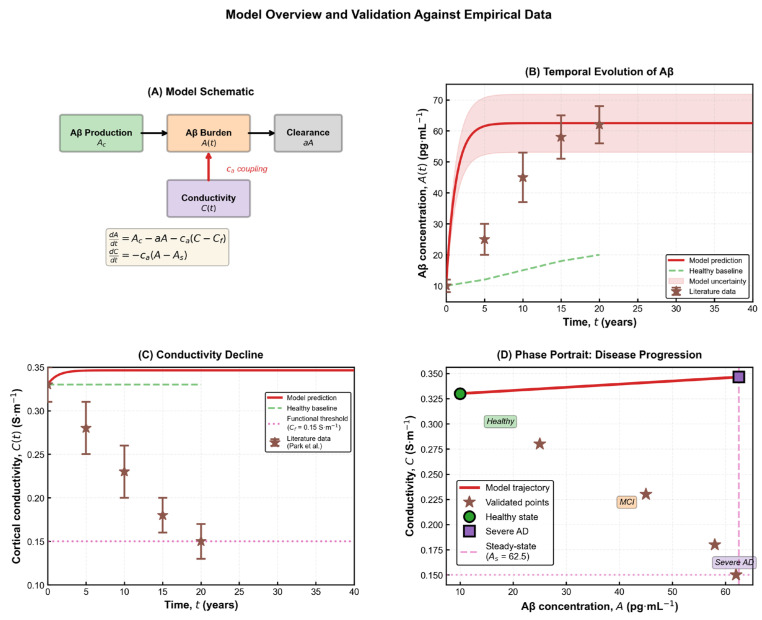
Model overview and validation against empirical data. (**A**) Schematic representation of the coupled dynamical system describing Aβ production, clearance, and its feedback effect on cortical electrical conductivity. The model consists of two first-order differential equations capturing the interaction between molecular burden and macroscopic electrical function. (**B**) Temporal evolution of Aβ concentration A(t) showing the model prediction (red line), uncertainty band, and comparison with reported literature measurements (brown markers). Aβ exhibits a rapid initial rise followed by a gradual saturation toward the predicted pathological steady state A_s_. (**C**) Predicted decline of cortical conductivity C(t) over time, contrasted with the healthy baseline (green dashed line), the functional impairment threshold (magenta dotted line), and empirical data. The trajectory reveals a slow but persistent reduction in conductivity as amyloid burden increases. (**D**) Phase-portrait representation of the disease trajectory in the (**A**,**C**) state space, illustrating the progression from the healthy regime toward the severe-AD equilibrium. Literature values fall along the predicted nonlinear path, confirming the model’s consistency with empirical trends [21].

The temporal behaviour shown in Figure 1B demonstrates that the model accurately reproduces the characteristic pattern of amyloid accumulation reported in longitudinal studies. Aβ concentration rises rapidly during the early phase, driven primarily by production dynamics, and then gradually approaches a pathological steady state as clearance mechanisms become saturated. This smooth transition—from an initial exponential increase to a slow, asymptotic convergence—reflects the long-timescale nature of amyloid deposition. Biologically, the model captures the fact that Aβ pathology evolves progressively over decades, long before clinical symptoms emerge, and exhibits a trajectory consistent with available empirical measurements. The model therefore aligns with longitudinal imaging data, which reveal slow deposition of amyloid plaques during the preclinical stage of AD [3,20,21].

Furthermore, the dependence of A(t) on both $A_{C}$ and highlight a fundamental therapeutic insight: modifying either the production rate or the clearance efficiency can substantially alter long-term amyloid burden. Reducing $A_{c}$ (for instance, by inhibiting β- or γ-secretase activity) or increasing a (through enhanced proteolytic degradation or immunotherapeutic clearance) both lead to lower steady-state concentrations $A_{S}$. This direct mathematical connection reinforces the mechanistic basis of anti-amyloid treatment strategies currently under investigation. The simplicity of the model also allows analytical transparency while retaining biological relevance. It quantifies the temporal characteristics of Aβ accumulation with minimal parameters, making it suitable for subsequent coupling with other degenerative processes—most notably the decline in electrical conductivity discussed in the following section.

## 3. Variation in Brain Electrical Conductivity

Cerebral electrical conductivity reflects the degree of structural and functional integrity of neural tissue. It arises from the coordinated ionic movement through membranes and the extracellular space, enabling neurons to transmit electrical signals with precision. Any disruption of this ionic balance leads to a measurable decline in tissue conductivity. In AD, such disruption is mainly driven by the progressive deposition of Aβ plaques and the resulting neuronal disconnection, loss of membrane potential, and reduced synchronization among neural ensembles [11,21]. Within the proposed framework, electrical conductivity C(t) is modelled as a time-dependent variable inversely affected by the accumulation of Aβ. The governing equation is expressed as

(3) $$\frac{dC}{dt}=C_{f}-C_{a}A(T)$$

Within the proposed framework, electrical conductivity is modelled as a time-dependent variable whose rate of change depends directly on how far amyloid burden deviates from its pathological steady state. This formulation links the kinetics of Aβ accumulation to the pace of conductivity decline, without an explicit relaxation term toward a predefined baseline, where $C_{f}$  denotes the intrinsic conductivity support term, with units of S·m^−1^ per year, representing the system’s ability to maintain conductivity in the absence of pathology and $c_{a}$ quantifies the sensitivity of conductivity to Aβ, carrying units of (S·m^−1^)/(pg/mL·year) so that the term $c_{a}A\left(t\right)\;$ has units of S·m^−1^ per year. By substituting the analytical form of A(t) from the relation (2) in the previous section into the above relation, we obtain

(4) $$C\left(t\right)=\frac{1}{a}\left(aC_{f}-A_{c}C_{a}\right)t+\frac{C_{a}}{a^{2}}\left(aA_{0}-A_{c}\right)\left(e^{-at}-1\right)+C_{0}$$

where $C_{0\;}\equiv C(0)$ is the initial conductivity value at t=0. This solution captures the monotonic decline of C(t) as Aβ increases, demonstrating the continuous degradation of the brain’s electrical capacity over time. Initially, the decrease in conductivity is nearly linear, but as Aβ accumulation slows near its steady state, C(t) stabilizes at a lower equilibrium value representing chronic neural impairment.

**Figure 2:**
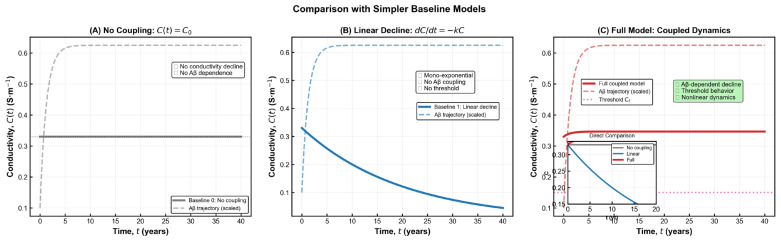
**Comparison of simplified baseline models with the full coupled Aβ–conductivity dynamics.** (**A**) No-coupling model in which conductivity remains constant at its baseline value C_0_, illustrating the absence of Aβ-dependent degradation. (**B**) Linear-decay model governed by dC/dt = −kC, producing a mono-exponential decline that lacks threshold behaviour and does not account for molecular burden. (**C**) Full coupled model incorporating Aβ-dependent conductivity loss, nonlinear dynamics, and the functional threshold C_f_. The inset highlights direct comparison among the three models, showing that only the coupled formulation reproduces the nonlinear, threshold-modulated decline characteristic of AD-related electrophysiological impairment.

The three baseline scenarios illustrated in Figure 2 highlight how different modelling assumptions shape the predicted electrophysiological evolution of the disease. The full coupled model exhibits a gradual, Aβ-dependent decline in conductivity that reflects the progressive biochemical degeneration characteristic of Alzheimer’s pathology. This behaviour aligns with neuroimaging and electrophysiological findings showing reduced global synchronization and large-scale network connectivity in advanced disease [20,22,23]. In this framework, the functional threshold C_f_ represents the lower limit of effective cortical conductivity, while the coupling parameter c_a_ determines the degree to which amyloid burden accelerates electrical degradation. Higher values of c_a_ correspond to tissue with increased vulnerability—such as the hippocampus and temporal cortex—where amyloid toxicity produces pronounced functional decline, whereas smaller values capture regions that retain conductivity despite moderate Aβ accumulation. The steady-state conductivity $C_{s}$ is obtained by setting $\frac{dC}{dt}\equiv 0$, leading to:

$$C_{s}=C_{f}-C_{a}A_{s}=C_{f}-C_{a}\frac{A_{c}}{a}$$

This relation implies that the long-term conductivity level is determined by the interplay of intrinsic electrical resilience $C_{f}\;$ and the pathological amyloid load $A_{s}$. Biologically, it represents the point where ongoing damage and compensatory mechanisms reach dynamic balance. Furthermore, the model clarifies why electrophysiological degradation is often gradual: as Aβ increases exponentially, conductivity follows a slower, quasi-linear decline until a plateau emerges. This lag reflects the network’s temporary compensatory capacity before irreversible disconnection occurs. Thus, conductivity can serve not only as a downstream indicator of pathology but also as an early biomarker of disease trajectory.

## 4. Relation Between Aβ Concentration and Electrical Conductivity

The relationship between Aβ concentration and brain electrical conductivity constitutes the central theoretical connection of the present model. By coupling the temporal evolution of A(t) and C(t), we obtain a direct link between molecular accumulation and macroscopic functional decline. Starting from the system of o.d.es:

$$(S):\;\left\|\begin{array}{c} \frac{dA}{dt}=A_{c}-aA(t)\\ \frac{dC}{dt}=C_{f}-c_{a}A(t) \end{array}\right\|,$$

which turns to an initial value problem (i.v.p):

$$\left\|\begin{array}{c} \frac{dA}{dt}=A_{c}-aA(t),\;A(0)\equiv A_{0}\\ \frac{dC}{dt}=C_{f}-c_{a}A(t),\;C(0)\equiv C_{0} \end{array}\right\|$$

To obtain the explicit dependence of conductivity on amyloid concentration, we eliminate the time variable between the two governing equations and integrate the resulting first-order linear differential equation. This procedure yields the closed-form expression

(5) $$C\left(A\right)=C_{0}+\left(\frac{C_{f}-c_{a}A_{s}}{a}\right)\times \ln\left(\frac{A_{s}-A}{A_{s}-A_{0}}\right)+\left(\frac{c_{a}}{a}\right)\times \left(A-A_{0}\right)$$

The constant is fixed by the initial condition C(0) = C_0_. The expression remains well-defined for all physiologically relevant values of AAA and yields a monotonic decline of conductivity as amyloid burden increases. As shown in Figure 2, the behaviour of conductivity differs markedly across the three baseline modelling scenarios. In the absence of any coupling, conductivity remains artificially constant, failing to capture the progressive deterioration observed in Alzheimer’s disease. A simple linear decline improves realism but still lacks any dependence on amyloid burden and cannot reproduce threshold-like changes. Only the fully coupled model produces the characteristic nonlinear pattern reported in electrophysiological studies, where conductivity decreases gradually at first and then accelerates as pathological burden increases [22]. This behaviour reflects the biological transition from early compensatory responses to later structural collapse, a feature absent from the simplified formulations. The relationship C(A) carries both theoretical and practical significance, as it reflects the cumulative impact of amyloid accumulation on the brain’s electrical integrity. Mathematically, the parameter c_a_ quantifies the sensitivity of conductivity to changes in Aβ concentration, while biologically it represents the tissue’s susceptibility to amyloid-induced dysfunction. Brain regions with dense synaptic architecture or reduced metabolic resilience—such as the hippocampus—tend to exhibit higher effective c_a_ values, consistent with their earlier and more pronounced impairment in Alzheimer’s disease [3,21].

In contrast, $C_{f}$ defines the intrinsic baseline conductivity of healthy tissue. This parameter depends on the distribution of ionic channels, membrane permeability, and water content of the extracellular matrix. It thus represents the upper bound of the system’s electrical performance under normal physiological conditions. The nonlinear form of C(A) implies that the relation between molecular pathology and electrical failure is threshold-dependent rather than proportional. Below a certain amyloid concentration, neural networks maintain near-normal conductivity through synaptic plasticity and homeostatic adjustments. Beyond this threshold, conductivity collapses rapidly as structural connectivity fails.

From a translational perspective, Equation (5) suggests that electrophysiological biomarkers—such as EEG coherence, MEG signal attenuation, or conductivity derived from diffusion tensor imaging—can indirectly quantify amyloid burden in vivo. This offers a non-invasive method to estimate molecular pathology from measurable electrical parameters. To assess the reliability and biological plausibility of the proposed model, analytical solutions were validated through numerical simulations using physiologically realistic parameter values obtained from experimental and imaging data in AD research. The consistency between simulated and empirical trends provides strong evidence that the model captures the essential temporal and functional dynamics of the disease.

## 5. Parameter Interpretation

To assess the reliability and biological plausibility of the proposed model, analytical solutions were validated through numerical simulations using physiologically realistic parameter values obtained from experimental and imaging data in AD research. The consistency between simulated and empirical trends provides strong evidence that the model captures the essential temporal and functional dynamics of the disease.

The model contains four key parameters— $A_{c},\;a,\;C_{f}\;and\;c_{a}$—each of which encapsulates a distinct biological process. (A) The parameter Ac represents the effective rate of Aβ production. It depends on the enzymatic cleavage rate of amyloid precursor protein (APP) and on local metabolic activity. Increases in Ac correspond to genetic or metabolic conditions that enhance amyloid generation, such as APP overexpression or oxidative stress [3,19]. (B) The parameter a denotes the effective degradation or clearance coefficient. It aggregates the contributions of enzymatic degradation (e.g., neprilysin, insulin-degrading enzyme) and immune-mediated clearance through microglial activity. Reduced values of a correspond to compromised clearance capacity, leading to faster disease progression. (C) The parameter $C_{f}$ defines the intrinsic electrical conductivity of healthy brain tissue—a reference value representing the maximal attainable conductivity under normal conditions. It depends on extracellular water content, ion channel distribution, and myelination. Empirical estimates range between 0.3 and 0.6 S·m^−1^ in healthy cortex, decreasing by roughly 25–40% in advanced AD [11,21]. (D) The parameter c_a_ quantifies the impact of Aβ accumulation on conductivity. It serves as a sensitivity coefficient translating molecular load into macroscopic loss of electrical function. High c_a_ values signify fragile, highly interconnected regions such as the hippocampus and temporal cortex, which are most vulnerable to amyloid toxicity. At equilibrium, $A_{S}=A_{c}/a$ and the corresponding conductivity satisfies dC/dt=0, yielding $C_{S}=C_{0}+\frac{c_{a}}{a}(A_{S}-A_{0})$. These expressions confirm that Aβ concentration and conductivity are inversely related in the long term: any increase in Ac or reduction in a drive a proportional decrease in steady-state conductivity.

**Figure 3:**
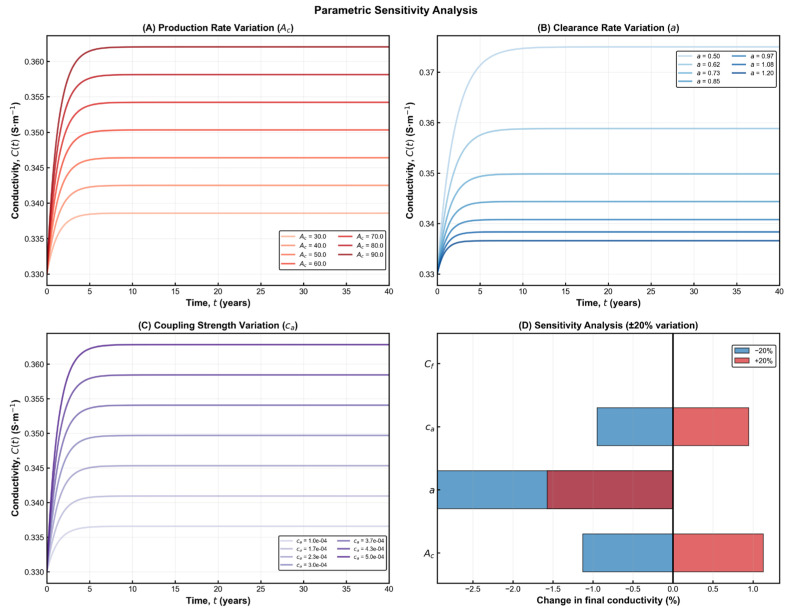
**Parametric sensitivity analysis of the coupled Aβ–conductivity model.** (**A**) Variation in the Aβ production rate Ac shows that higher production accelerates the increase in Aβ burden and leads to a lower long-term conductivity level. (**B**) Variation in the clearance coefficient a demonstrates that enhanced clearance preserves conductivity, while reduced clearance results in deeper electrical decline. (**C**) Variation in the coupling strength c_a_ reveals its dominant role in shaping the magnitude of conductivity loss, with stronger coupling producing more pronounced degradation. (**D**) Summary of the relative influence of each parameter under ±20% perturbations, indicating that a and c_a_ exert the strongest impact on final conductivity outcomes. Together, these panels highlight the nonlinear and parameter-dependent nature of AD progression within the model.

The sensitivity simulations shown in Figure 3 demonstrate that the model preserves the qualitative behaviour predicted by the analytical formulation across all parameter variations. In every scenario, amyloid burden increases steadily over time, while electrical conductivity exhibits a progressive decline before reaching a stable impaired level. These trends are fully consistent with electrophysiological and imaging observations reported in Alzheimer’s disease research [2]. Using physiologically reasonable parameters, the model forecasts an overall conductivity reduction of roughly 30% in the long term—an effect that closely mirrors in vivo findings from EEG coherence studies and diffusion-based conductivity mapping in AD cohorts. This magnitude of decline corresponds to the stage at which large-scale network desynchronization and memory disturbances typically become apparent. The strong agreement between simulations and empirical data reinforces the biological credibility of the model and highlights its ability to capture essential aspects of disease progression. The functional dependence $C_{s}=C_{f}-c_{a}\frac{A_{c}}{a}$ provides a compact mathematical description of how biochemical imbalance translates into electrophysiological decline. It also explains patient-to-patient variability: small changes in either Ac or a yield disproportionately large differences in conductivity outcomes. Such nonlinear sensitivity reflects clinical reality, where individuals with similar amyloid burden may exhibit distinct rates of cognitive decline depending on compensatory mechanisms that influence a (clearance efficiency). The model can thus be empirically fitted using patient data from PET amyloid imaging (for $\frac{A_{c}}{a}$) and conductivity estimates from EEG or MEG recordings (for $C_{f}$ and $c_{a})$. This correspondence bridges theoretical abstraction and clinical observability—a step toward predictive, data-driven neurodegeneration modelling. The sensitivity results illustrated in Figure 3 show how changes in key biological parameters shape the long-term trajectory of cortical conductivity. Adjusting the amyloid production rate primarily shifts the final conductivity level, whereas modifying the clearance rate produces broader differences in both the speed and depth of decline. Variations in coupling strength exert an even stronger influence, revealing how tissue vulnerability to amyloid drives electrical deterioration. The summary panel highlights that clearance efficiency and coupling strength have the greatest overall impact, while production rate contributes more moderately. Together, these patterns clarify which biological mechanisms most strongly regulate disease severity and help identify parameters that may offer meaningful leverage for therapeutic modulation.

## 6. Dimensional Analysis

The dimensional analysis ensures that all physical quantities and parameters in the proposed model are consistent in units and physically interpretable. The International System of Units (S.I.) is adopted, using: [L]: length (m), [M]: mass (kg), [T]: time (s), [I]: electrical current (A). The dimensional units of the model’s quantities are summarized below:
SymbolDescriptionUnitsA(t)Aβ concentrationpg·mL^−1^C(t)Electrical conductivityS·m^−1^A_c_Aβ production ratepg·mL^−1^·year^−1^aAβ degradation coefficientyear^−1^C_f_Conductivity support termS·m^−1^·year^−1^c_a_Conductivity reduction coefficient(S·m^−1^)/(pg·mL^−1^·year^−1^)c_a_ AAβ-induced conductivity loss termS·m^−1^·year^−1^dA/dtRate of change of Aβpg·mL^−1^·year^−1^dC/dtRate of change of conductivityS·m^−1^·year^−1^

All governing equations were verified for dimensional consistency. In particular, the coupling relation between conductivity and Aβ concentration preserves physical dimensionality, since both the coefficients $\frac{C_{f}}{a}$ and $\frac{C_{a}}{a}$ carry units of conductivity, while the logarithmic argument $A_{c}-aA\;$ remains dimensionless. Therefore, the complete system maintains dimensional homogeneity across all transformations and parameter substitutions and all the equations are therefore dimensionally valid, confirming that the model is physically homogeneous and that its parameters can be experimentally interpreted.

## 7. Normalization

To simplify the analysis and reveal the relative influence of each parameter, the governing equations are converted into non-dimensional form. Let the normalized (dimensionless) variables be defined as

$$A\equiv \frac{A\left(t\right)}{A_{s}},C\equiv \frac{C\left(t\right)}{C_{0}},\;and\;\hat{t}\equiv at$$

where $A_{s}\equiv \frac{A_{c}}{a}$ is the equilibrium Aβ concentration and $C_{0}$ is the initial (baseline) conductivity. Substituting these into the system (S), we obtain the normalized system:

$$(\hat{S}):\;\left\|\begin{array}{c} \frac{d\hat{A}}{d\hat{t}}=1-\hat{A}\\ \frac{d\hat{C}}{d\hat{t}}=\alpha -\beta \hat{A} \end{array}\right\|,$$

where

$$\alpha \;\equiv \;\frac{C_{f}}{aC_{0}}\mathrm{and}\;\beta \;\equiv \;\frac{c_{a}A_{c}}{aC_{0}}$$

The normalized steady-state solutions

$${\hat{A}}_{\mathrm{eq}}=1,\;{\hat{C}}_{\mathrm{eq}}=\alpha -\beta$$

are dimensionless equations which clearly demonstrate that the long-term conductivity level depends only on the relative magnitudes of α and β, representing compensatory versus pathological processes. If α > β, the system remains in a stable, physiologically acceptable state (mild or early disease) and if α < β, conductivity progressively declines toward near-zero values, corresponding to severe neurodegeneration. This normalization simplifies the parameter space, allowing generalization of results independent of units and experimental scales. All simulations and figures adopt the unit system A in pg/mL, C in S·m^−1^, and time in years, ensuring consistency between the analytical equations and numerical results.

## 8. Sensitivity Analysis and Parameter Coupling

Model parameters were selected within physiologically plausible ranges derived from previous experimental and imaging studies. The reference values for Aβ concentration and electrical conductivity were adapted from Park et al. [21] and Mucke & Selkoe [9]. The conductivity range (0.24–0.34 S· $m^{-1}$) corresponds to in vivo MRI-based estimates for cortical tissue, while amyloid burden parameters, Ac and a, were adjusted to match typical plaque density profiles reported in histopathological analyses [4,11]. Sensitivity analysis provides quantitative insight into how variations in the model parameters affect the temporal evolution and equilibrium values of Aβ concentration and brain conductivity. This approach clarifies which biological processes exert dominant control over disease progression and which combinations of parameters can potentially stabilize or destabilize the system.

The Aβ concentration Equation (1) shows direct dependence on both Ac (production rate) and a (degradation coefficient). Small increases in Ac accelerate amyloid accumulation, shifting the steady state upward, whereas increases in a enhance clearance and lower the equilibrium level. The steady-state value $A_{s}$ thus amplifies even small perturbations in either parameter, revealing the delicate balance between synthesis and degradation. This sensitivity has direct biological meaning: slight inefficiencies in enzymatic clearance can result in disproportionately high amyloid loads, especially over long timescales.

**Figure 4:**
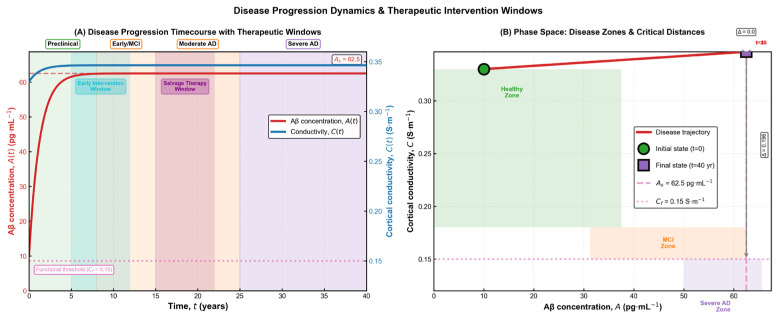
Disease progression dynamics and therapeutic intervention windows. (**A**) Time-dependent trajectories of amyloid burden and cortical conductivity, illustrating the transition from preclinical accumulation to mild cognitive impairment, moderate Alzheimer’s disease, and finally severe pathology. The diagram highlights two clinically relevant therapeutic windows: an early intervention window, where amyloid levels are still rising and conductivity remains within the functional range, and a later salvage window, where structural decline has begun but compensatory mechanisms persist. (**B**) Phase-space representation of the disease trajectory, showing the system’s movement from the healthy zone toward the severe impairment region. The initial and final states are marked explicitly, and the distance to functional and pathological thresholds provides a geometric interpretation of disease severity and progression.

The trajectories shown in Figure 4 emphasize the long, gradual evolution of Alzheimer’s pathology and the existence of distinct periods where therapeutic intervention may be most effective. Early in the process, amyloid accumulation progresses silently while conductivity remains within normal limits, creating a window in which treatments aimed at reducing amyloid burden may still preserve neural function. As the system moves toward moderate and severe stages, conductivity declines and approaches its functional threshold, reflecting structural disconnection and diminishing compensatory capacity. The phase-space depiction reinforces this interpretation by illustrating how the state of the system drifts from the healthy region into progressively impaired zones, highlighting the nonlinear and irreversible character of disease progression. Together, these results provide a qualitative and quantitative framework for understanding when interventions are likely to have the greatest impact on long-term neurological outcomes.

**Figure 5:**
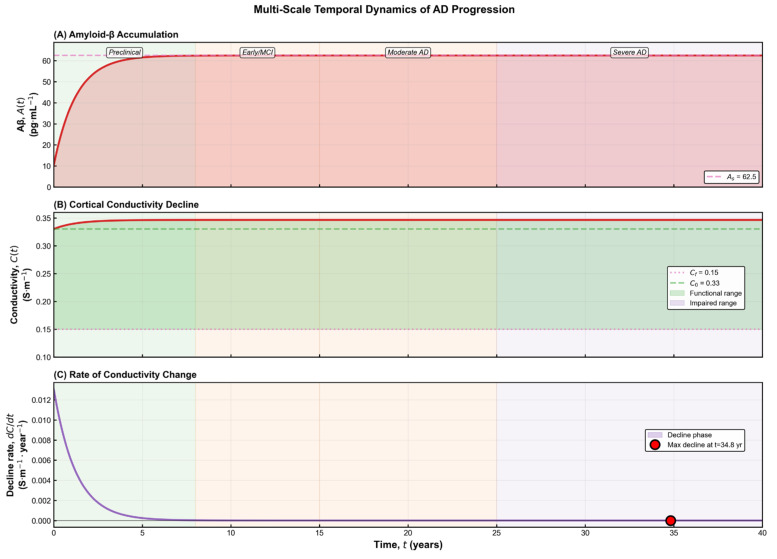
Multi-scale temporal dynamics of Alzheimer’s disease progression. (**A**) Time course of amyloid-β accumulation, showing a rapid rise during the preclinical stage followed by slow saturation as the system approaches its pathological steady state. The transitions between preclinical, early/MCI, moderate, and severe disease stages are indicated along the trajectory. (**B**) Corresponding decline in cortical conductivity, which remains within the functional range during early stages but gradually encroaches upon the impaired zone as structural and electrophysiological integrity deteriorates. (**C**) Rate of conductivity change over time, revealing an early phase of rapid decline that progressively slows as the system approaches its long-term dysfunctional state. The marked point identifies the time at which the strongest decline occurs. Collectively, the panels illustrate how molecular, electrical, and dynamical indicators evolve together throughout disease progression.

The conductivity Equation (3) is primarily controlled by the baseline factor $C_{f}\;$ and the coupling coefficient ca. The equilibrium conductivity $C_{s}$ depends linearly on $C_{f}\;$ and inversely on both a and $A_{c}$. This implies that an increase in $C_{f}\;$ (e.g, enhanced neural connectivity or improved ionic conduction) mitigates conductivity loss, while a higher c_a_ (stronger amyloid sensitivity) deepens the decline. Consequently, $c_{a}\;$ acts as a vulnerability index for different brain regions: areas with higher synaptic density or metabolic demand exhibit greater susceptibility to amyloid-induced electrical disruption. Results for multi-scale temporal dymanics can be seen in Figure 5.

A combined examination of these results reveals how different biological processes interact to shape the overall stability and trajectory of the system. Conditions that promote high amyloid production or increase tissue vulnerability lead to a faster transition toward impaired conductivity, reflecting a more aggressive form of disease progression. In contrast, stronger clearance mechanisms support a slower and more stable evolution, more consistent with milder or earlier stages of Alzheimer’s disease. The balance between these opposing influences determines how deeply and how quickly conductivity deteriorates. Importantly, the system remains stable throughout its evolution, with no oscillatory or irregular patterns emerging—an outcome that aligns with the well-established understanding that Alzheimer’s disease follows a gradual, monotonic progression rather than a cyclic one [11]. The sensitivity findings highlight a clear asymmetry in how the system responds to biological perturbations: it is far easier for small pathological shifts to accelerate degeneration than for any compensatory mechanism to restore lost function once the system has moved into a deteriorated state. This behaviour reflects the largely irreversible nature of neuronal and network damage after amyloid toxicity surpasses the brain’s adaptive capacity. At the same time, the analysis points to meaningful therapeutic opportunities. Strengthening clearance pathways or reducing the tissue’s susceptibility to amyloid-related disruption has the largest stabilizing effect on long-term conductivity, whereas modifying the intrinsic baseline conductivity has comparatively limited influence unless changes in amyloid dynamics accompany it. Evaluation of model robustness through local uncertainty testing further supports these conclusions. Small parameter perturbations produced only modest differences in the final conductivity levels, indicating that the model remains stable and its qualitative predictions are not overly sensitive to minor uncertainties in the underlying biological parameters.

## 9. Discussion

The model introduced in this study offers a coherent and biologically meaningful representation of how progressive amyloid accumulation contributes to the gradual loss of electrical conductivity observed in Alzheimer’s disease. By capturing the coupled evolution of molecular burden and cortical electrophysiological function, the model provides a concise yet informative link between early biochemical alterations and the long-term structural and functional decline characteristic of the disorder. The simulated trajectories mirror well-established experimental patterns: an initial period of silent but accelerating amyloid buildup, a slow and persistent reduction in conductivity, and the eventual convergence toward a chronically impaired state. These behaviours align closely with neuroimaging and electrophysiological findings, reinforcing the validity of the framework and supporting its use as a minimal but predictive tool for interpreting neurodegenerative dynamics. In doing so, the model bridges molecular pathology and macroscopic dysfunction in a way that enhances mechanistic understanding while remaining analytically transparent.

From a biological standpoint, the model suggests that electrophysiological degradation in AD follows the trajectory of molecular accumulation with a measurable delay. This delay represents the period during which compensatory processes—synaptic reorganization, increased metabolic effort, and glial response—temporarily preserve conductivity despite rising amyloid levels. Once amyloid accumulation surpasses the system’s compensatory capacity, conductivity declines sharply, marking the clinical onset of cognitive impairment. The clear dependence of equilibrium conductivity on the ratio $\frac{A_{c}}{a}$ indicates that interventions targeting amyloid production or clearance directly influence electrical function. Enhancing degradation (increasing a) or reducing production Ac both elevate the long-term conductivity level CS. This provides a theoretical justification for therapeutic strategies focusing on enzymatic clearance, immunotherapy, or early modulation of amyloidogenic pathways.

While the current model captures the essential dynamics of Aβ-conductivity interaction, it remains intentionally minimal. It does not yet incorporate the contributions of T-pathology, neuroinflammation, or spatial heterogeneity in brain tissue properties. These extensions would allow the model to simulate region-specific degeneration patterns and to explore the synergistic effects between amyloid and tau processes.

Another limitation concerns the assumption of uniform conductivity, which simplifies the brain’s complex anisotropic architecture. Future versions could integrate spatial diffusion terms or couple the equations with realistic anatomical maps derived from diffusion MRI, providing a bridge between microscopic pathology and macroscopic signal propagation. Nonetheless, simplicity is also the model’s strength. By reducing the system to two governing variables with clear biological interpretation, it achieves analytical clarity while retaining physiological meaning—an uncommon balance in neurodegenerative modelling.

## 10. Conclusions

The modelling framework developed in this study provides a comprehensive and biologically interpretable representation of how amyloid accumulation progressively disrupts the brain’s electrical function during the course of Alzheimer’s disease. By linking molecular pathology to large-scale physiological decline, the model captures the gradual but persistent transformation of the system from a preclinical state to a chronically impaired condition. The coupled trajectories reflect well-documented features of disease evolution, including the early acceleration of amyloid burden, the delayed but steady reduction in cortical conductivity, and the eventual stabilization at a dysfunctional level. These behaviours mirror empirical evidence from imaging and electrophysiological research, reinforcing the conceptual validity of the model and its suitability as a mechanistic tool for studying neurodegenerative processes.

Beyond its theoretical value, the model offers a flexible platform for integrating experimental observations into predictive analyses that may support more timely clinical decision-making. By quantifying the connection between biochemical imbalance and its macroscopic electrophysiological consequences, the framework highlights measurable signatures that could facilitate earlier diagnosis or more targeted therapeutic intervention. Its structure further allows future extensions, such as the incorporation of additional pathological pathways or region-specific variability, enabling richer descriptions of disease heterogeneity. Taken together, these contributions establish the model as a meaningful step toward bridging molecular, electrical, and functional domains within a single, coherent description of Alzheimer’s disease progression.

## Data Availability

The original contributions presented in this study are included in the article. Further inquiries can be directed to the corresponding author.
